# The Interplay between Public Health, Well-Being and Population Aging in Europe: An Advanced Structural Equation Modelling and Gaussian Network Approach

**DOI:** 10.3390/ijerph18042015

**Published:** 2021-02-19

**Authors:** Mirela Cristea, Graţiela Georgiana Noja, Cecilia-Nicoleta Jurcuţ, Constantin Ştefan Ponea, Elena Sorina Caragiani, Alin Viorel Istodor

**Affiliations:** 1 Faculty of Economics and Business Administration, Department of Finance, Banking and Economic Analysis, University of Craiova, 200585 Craiova, Romania; mirelas.cristea@gmail.com; 2 Faculty of Economics and Business Administration, Department of Marketing and International Economic Relations, West University of Timisoara, 300115 Timisoara, Romania; 3 Faculty of Economics and Business Administration, Department of Management, West University of Timisoara, 300115 Timisoara, Romania; cecilia.jurcut@e-uvt.ro; 4 Faculty of Legal, Economic and Administrative Sciences, Department of Economics, Spiru Haret University, 200580 Craiova, Romania; ponea.stefan@yahoo.com; 5 Faculty of Economic and Business Administration, Doctoral School of Economics, University of Craiova, 200585 Craiova, Romania; sorina_caragiani@yahoo.com; 6 First Department of Surgery, Second Discipline of Surgical Semiology, Victor Babes University of Medicine and Pharmacy, 300041 Timisoara, Romania; istodor.alin@umft.ro

**Keywords:** econometric modelling, health, aging, well-being, European Union

## Abstract

Given the COVID-19 pandemic crisis that has deeply affected the health and well-being of people worldwide, the main objective of this paper was to explore the existing relationship between health, welfare, and population aging until the pandemic burst, on the basis of two distinctive groups of European Union (EU) countries, namely, the old and the new member states. The methodological endeavor was based on two advanced econometric techniques, namely, structural equation modelling and network analysis through Gaussian graphical models, applied for each group of EU countries, analyzed during the period of 1995–2017. The main results revealed significant differentiation among the new and old EU countries as follows: public health support was found to have a positive impact on healthy aging and well-being of older people, on other social determinants, and on people’s perceived good and very good health; overall, significant influences were revealed in terms of the aging dimensions. The main implications of our findings relate to other researchers as a baseline comparison with the existing situation before the COVID-19 pandemic outbreak, but also to policymakers that have to rethink the public health allocations, both in old and new EU member states, in order to endorse the aging credentials, underpinning a successful and healthy integration of the elderly within all life dimensions.

## 1. Introduction

The aging population phenomenon, manifested by the simultaneous action of an increasing life expectancy and downsized birth rate [1,2], is affecting countries worldwide, with multiple implications on the economy [3,4,5], at a social level [6], and in terms of the health and well-being [7,8,9] dimensions. The high pressure of population aging is now facing unpredictable challenges caused by the coronavirus disease 2019 (COVID-19) pandemic crisis that has taken a toll on health expenditures for all the demands, especially those of the elderly people [10]. The COVID-19 pandemic challenges have a major impact also on the well-being of people, with multiple unexpected consequences upon the economic and social life, thus reinforcing an ongoing need for a comprehensive analysis of the determinants of aging, widely shaped by the new socio-economic conditions.

At the same time, the aging phenomenon can represent both an opportunity and a threat [11]. The opportunities derived from living longer are strongly dependent on a healthy life [7]. Thus, elderly people can enjoy life, being involved in social (volunteering), cultural, or political activities; grandchild care activities; and, in the time of COVID-19 outbreak, even “helping society to fight against the pandemic (…) by helping communities in different ways” [10] (p. 1). These coordinates were enclosed into “the active ageing” strategy [12], which has been transposed at the European Union (EU) level since 2012 [13,14]. Active aging strategy is accompanied by a “healthy ageing” strategy [12]. Moreover, during the COVID-19 pandemic crisis, if the life of seniors (65+) also suffering from “a decrease in physical or mental capacity” [7] (p. 2145) would not have been supported by health-promoting conditions, the impact on individuals and society would have been worse.

In this regard, the World Health Organization (WHO) declared the period 2020–2030 the ”Decade of Healthy Ageing” [15], on the basis of 10 priorities [16] (pp. 3–15), including innovation and change platforms, sustaining policies/strategies, gathering worldwide data on healthy aging available for policymakers and scientists in order to evaluate and differentiate among countries, determining the new needs of senior citizens, appraising the countries’ health systems/services in order to align and support them, introducing a long-term care system in every country, including specialized staff, policy to increase public awareness against “ageism”, “understanding the costs and opportunities”, and age-friendly cities/communities. On the basis of these strategies and new challenges, we find that the public health support for ensuring a healthy life of older people (65+) is essential for providing active and healthy aging [3,5,17]. Changes in health behavior (diet and physical activity) and changes in the work environment (i.e., type of work, employment sector, occupational health and safety regulations, etc.) are definitely altogether associated with physical fitness and health.

In terms of these challenging backgrounds, the current study undertook several important issues in health economics and configured an ambitious theoretical and empirical research approach to strengthen the knowledge in this scientific field. Hence, the general objective of our paper was to assess the implications of public health support/services on what it is perceived by people as good and very good health and the aging dimensions, at the EU level (distinctively in the old and new EU countries), under the impact of selected social dimensions (poverty and health conditions of older people, education, and income).

The paper entails significant contributions to the literature by offering a comprehensive perspective on the interlinkages between several important fundamentals of population ageing and public health support through the lens of an advanced empirical analysis. It therefore presents robust evidence on a causal relationship between government health expenditures, hospital services, and the good health and well-being of the elderly population (aged 65+), with spillover effects on the aging dimensions.

The main research directions are, jointly, as follows: (i) to assess the direct implications of public health support (reflected by health government expenditures and hospital services) upon selected social variables (people’s income; health of elderly persons aged 65 years and older; poverty rate of older people; education components, levels 3–8 and tertiary); (ii) to estimate how the cumulative effects of the above associations could impact the perceived good and very good health (as one of the 17th Sustainable Development Goals—SDG, namely, Goal 3 “Good health and well-being”); (iii) and to appraise the overall impact of health and other social dimensions upon aging credentials, namely, birth rate and life expectancy (the latter also being a SDG indicator, included in Goal 3). The methodological endeavor is based on two advanced econometric techniques, namely, the structural equation modelling (SEM)—latent class analysis, and network analysis through Gaussian graphical models (GGMs) for each group of countries (old and new EU countries). We chose to analyze the two main groups of EU countries, given the dissimilarities among them [4,14]. The data cover the period 1995–2017 and were gathered from the Eurostat database [18].

The rest of the paper is structured as follows. Section 2 entails a brief synthesis of literature underpinnings, related to the main findings on health and other social implications on population aging in different economies. Further, in Section 3, introduce the dataset used for our empirical analysis and the methodology applied, which is followed by the main results and the discussion presented in Section 4. Finally, concluding remarks are accompanied by policies and strategies recommended for each considered group of countries, the old and new EU member states (MSs).

## 2. Brief Literature Review

The health-enhancing measures have a powerful impact on life expectancy at birth and on the lifespan [12]. However, since birth rates and fertility rates have decreased, the old dependency ratio (the share of people over 65 years of age/people 15–64 years of age, population at the working age) has increased in the majority of countries around the world, from 8.59% globally in 1960 to 13.58% in 2018 [19]. Thus, nowadays, the social public health support system has suffered, due to plummeting healthcare and social contributions paid by the ever-shrinking working age population. This allotment of funds is particularly dedicated to older people, whose need for healthcare activities is far larger than those of the young [7,20,21,22,23]. This applies also to the recovery after a COVID-19 infection [10,23] (p. 3), which has shown that, no matter the origin, well-being, gender, or culture, that “health is regarded as the most important asset for active ageing”.

Cristea et al. [8] assessed the complex patterns of relationship between aging, public health support (government expenditures and services), and labor productivity through the insertion of the group of working people aged 55–64 years on the labor market. In this framework, Cristea et al. [8] underlined that the new EU countries (EU–13) were confronted with shortages in public health expenditures, while the old EU countries (EU-15) had to deal with issues related to the public hospital services. The authors [8] (pp. 13–14) recommended policies for the developing EU countries targeting “public health expenditure to focus on increasing birth rates and sustaining life expectancy since at the present the highest health needs/consumptions are for the elderly, both women and men; and public health expenditure for increasing the general health and perception of people, which would positively influence labor productivity”. In the case of developed EU countries, policymakers have to rethink public hospital services to enhance health and the people’s perception, “and thus, have them back public health expenditure to boost birth rate and offer support for child care, therefore indirectly sustaining birth rates and life expectancy” [8] (p. 14). The COVID-19 crisis put an unprecedented pressure on these systems, augmenting the existing shortcomings for the health sector, making even more requisite ongoing investigations in this regard, with an active network among governments that helps to adopt adequate policies [24].

As regards the relation between health and poverty, some findings [23,25] have shown that poverty plays a key role in people’s health, with “women, older adults, respondents with low education and income” being the most affected [26] (p. 16). Thus, the awareness of public authorities to counteract these consequences is paramount, especially in the developing countries [27], particularly Eastern European countries, where these relations are more evident [26,28]. Public health support within social policies is fundamental for the battle of poverty in front of older-age needs, as well as sustaining health from early age [28]. There are findings that have proven that for tackling the poverty of older people (over 60 years old), the most significant and perceived factor for this group of people is “adequacy of disposable income” rather than the “absolute income” that offers a higher strapping by the self-awarded health conditions, especially for women [29] (p. 1111).

Furthermore, significant positive interlinkages between work conditions/job quality and health conditions of older working groups (aged 55–64 years) were investigated at the EU level and have shown a positive impact on life expectancy and well-being, on the basis of the fact that “exposure to poor working conditions during midlife may correlate with health during retirement in later life” [30] (p. 60). Reviewing the relation between education, health and aging, research has shown that “people with a low level of education can expect to live six years less than those with a high level of education” [31] (p. 12). Therefore, education is improving people’s health/health perception and life expectancy, with this being the case for women rather than men, since they are more informed on health issues and health-enhancing habits at any given age [31,32,33,34]. Moreover, the positive impact of education on health is sustaining the active aging, which considerably varies across different countries [35]. Education can influence both people’s well-being, since “they experience higher utility at any age”, and life expectancy, explained by the fact that “they live longer due to their healthier behavior” [34] (p. 10). People’s health can be enhanced through a continuous encouragement and support of education from the governors, being proven that the more educated people are, the highest their health condition is, with direct impact on welfare—increased earnings due to easiness and motivation for having a job, thus reducing poverty [27].

Thereby, besides education, income represents a significant determinant of self-perceived health, which positively influences it—the more educated men and women are, the better the self-perceived health is, and vice versa [36]. Furthermore, income has an evidenced impact on health and life expectancy/longevity “through various clinical, behavioral, social, and environmental mechanisms”, mainly, through the financial possibility to access the medical care services [37] (p. 1). Khullar and Chokshi [37] grounded these findings on the fact that lower-income people are less included by the employers for health incentives/benefits that they grant to the employees, and thus this category of people has limited access to a wide range of healthcare services.

In summary, we would like to underline that the aging phenomenon is present across all countries around the world, being more visible in the EU countries, due to the highest old-age dependency ratio; furthermore, aging is influenced by many factors, such as public health support, poverty, education, and well-being, on the one hand; on the other hand, there are significant implications between aging and people’s perception on health. Facing the COVID-19 pandemic crisis, the healthcare systems are nowadays under a great deal of pressure, and the most affected components are the health of elderly people.

## 3. Data and Methodology

### 3.1. Data

On the basis of literature findings, our analysis focused on the following dimensions to reach its general objective: aging phenomenon, public health support and implications, and other social factors, which have been selected to reveal the implications of well-being (earnings), education, and poverty as main social dimensions of elderly people.

Thereby, the variables considered for our investigation are as follows [18]:

- aging indicators: “Crude birth rate (number of live births per 1000 people)” (BR); “Life expectancy at birth, total population (years)” (LE) (SDG indicator, Goal 3 “Good health and well-being”);
- health indicators: “Health government expenditure” (% of gross domestic product, GDP) (HGE); “Hospital services” (% of GDP) (HS); “Healthy life years in absolute value at 65—females (years)” (HLY_F); “Healthy life years in absolute value at 65—males (years)” (HLY_M); “Share of people aged 16+ with good or very good perceived health” (%) (SDG indicator, Goal 3 “Good health and well-being”) (%) (PGPH);
- other social representative indicators: “Annual net earnings of a two-earner married couple with two children (purchasing power standard)” (EARN); “Population with secondary, upper, post-secondary, and tertiary education (levels 3–8) (% of 15–64 aged years)” (EDU); “Tertiary education level 30–34 age group (% of the population aged 30–34)” (TE_30_34); At-risk-of-poverty-rate of older people, 65+ (%) (POV_R_65).

The indicators have been extracted from the Eurostat database [18] for the period 1995–2017.

**Figure 1:**
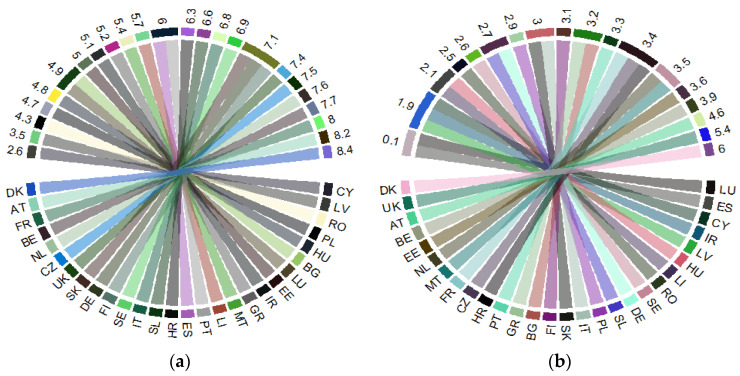
Public health support at the EU level: 2017: (**a**) “Health government expenditure” (% of gross domestic product, GDP) (HGE); (**b**) “Hospital services” (% of GDP) (HS). Source: Own process in R version 3.6.3. (R Foundation, Free Software Foundation’s GNU General Public License, Boston, MA, USA), based on Eurostat data [18]. Legend: AT—Austria; BE—Belgium; BG—Bulgaria; HR—Croatia; CY—Cyprus; CZ—Czech Republic; DK—Denmark; EE—Estonia; FI—Finland; FR—France; DE—Germany; GR—Greece; HU—Hungary; IR—Ireland; IT—Italy; LV—Latvia; LI—Lithuania; LU—Luxembourg; MT—Malta; NL—the Netherlands; PL—Poland; PT—Portugal; RO—Romania; SE—Sweden; SK—Slovak Republic; SL—Slovenia; ES—Spain; UK—United Kingdom. Value range: (**a**) 2.6–8.4 (% of GDP); (**b**) 0.1–6 (% of GDP).

Public health support (as share of GDP), reflected by HGE, on the one hand, across the EU countries in 2017 (Figure 1a) entails significant allocations in France, the Netherlands, Belgium, Austria, Denmark, and the United Kingdom (UK) (old EU countries), and the Czech Republic (among the new EU countries). On the other hand, in most of these countries, public hospital services (HS) are also at the highest levels, including Estonia (the new MS of the EU) (Figure 1b).

**Figure 2:**
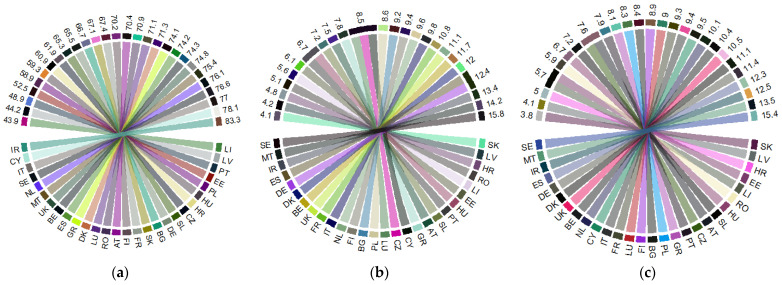
Health indicators in the EU in 2017: (**a**) “Share of people aged 16+ with good or very good perceived health” (%) (PGPH); (**b**) “Healthy life years in absolute value at 65—females (years)” (HLY_F); (**c**) “Healthy life years in absolute value at 65—males (years)” (HLY_M). Source: own process in R version 3.6.3. (R Foundation, Free Software Foundation’s GNU General Public License, Boston, USA), based on Eurostat data [18]. Legend: AT—Austria; BE—Belgium; BG—Bulgaria; HR—Croatia; CY—Cyprus; CZ—Czech Republic; DK—Denmark; EE—Estonia; FI—Finland; FR—France; DE—Germany; GR—Greece; HU—Hungary; IR—Ireland; IT—Italy; LV—Latvia; LI—Lithuania; LU—Luxembourg; MT—Malta; NL—the Netherlands; PL—Poland; PT—Portugal; RO—Romania; SE—Sweden; SK—Slovak Republic; SL—Slovenia; ES—Spain; UK—United Kingdom. Value range: (**a**) 43.9–83.3 (%); (**b**) 4.1–15.8 (years); (**c**) 3.8–15.4 (years).

The share of people (up to the age of 16 years) who perceive strong health (PGPH) (Figure 2a) is representative in several old EU countries (developed countries) (namely, Ireland Italy, Sweden, Netherlands, and the UK), compared with the new EU countries (developing countries) that perceive their health as being worse. Healthy life of older people (65+), both of women (Figure 2b) and men (Figure 2c), is significantly higher in the old EU MSs (the Nordic countries—Sweden, Denmark, as well as Ireland, Spain, and Germany,) than in the new EU countries that are at the bottom of the rankings (with the lowest level in the Slovak Republic).

**Figure 3:**
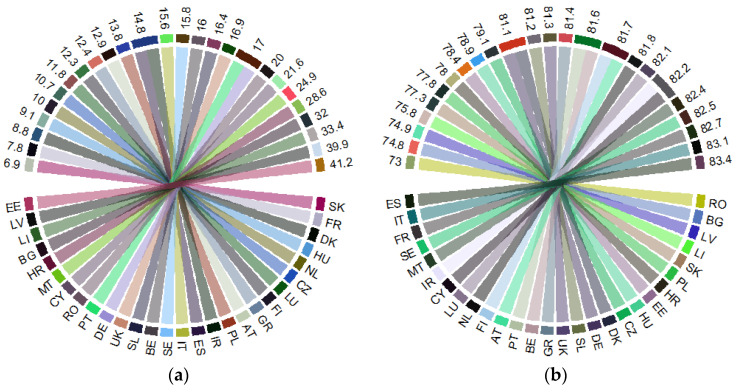

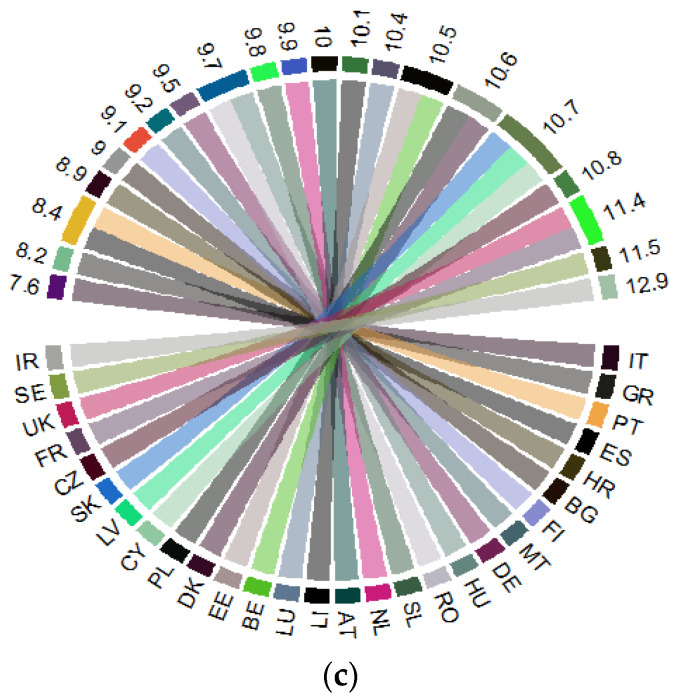
*Poverty 65+* and the *aging dimensions* within the EU, in 2017: (**a**) “At-risk-of-poverty-rate of older people, 65+ (%)” (POV_65); (**b**) “Life expectancy at birth, total population (years)” (*LE*); (**c**) “Crude birth rate (number of live births per 1000 people)” (BR). Source: own process in R version 3.6.3. (R Foundation, Free Software Foundation’s GNU General Public License, Boston, USA), based on Eurostat data [18]. Legend: AT—Austria; BE—Belgium; BG—Bulgaria; HR—Croatia; CY—Cyprus; CZ—Czech Republic; DK—Denmark; EE—Estonia; FI—Finland; FR—France; DE—Germany; GR—Greece; HU—Hungary; IR—Ireland; IT—Italy; LV—Latvia; LI—Lithuania; LU—Luxembourg; MT—Malta; NL—the Netherlands; PL—Poland; PT—Portugal; RO—Romania; SE—Sweden; SK—Slovak Republic; SL—Slovenia; ES—Spain; UK—United Kingdom. Value range: (**a**) 6.9–41.2 (%); (**b**) 73–83.4 (years); (**c**) 7.6–12.9 (number of live births per 1000 people).

By contrast, the new EU-13 countries are facing the highest share of older people (65+) living in poverty (namely, the Baltic States, Bulgaria, Croatia, Malta, and Cyprus) (Figure 3a), and the lowest, mostly in the old EU-15 countries (France, Denmark), along with the Slovak Republic (the low-ranking poverty of elderly people in the EU). *Aging dimensions* are at the highest level in the EU-15 countries, both as regards *life expectancy* (over 80 years, with Spain being 2017 the country with the highest LE at 83.4 years, followed by Italy, France, and Sweden) (Figure 3b), and birth rate (Ireland registering the highest value with 12.9 live births per 1000 people, followed by Sweden, the UK, and France) (Figure 3c). The opposite, the lowest levels of life expectancy, were registered in the EU-13 countries, with Romania being at the bottom of the ranks (with an average of 73 years). On the other hand, the lowest birth rates were registered in countries from the EU-15 group, with Italy, Greece, Portugal, and Spain at the bottom of the ranking, which reveal the high level of pressure on these countries, having the highest aged populations.

The summary statistics for each considered group of EU countries are presented in Table A1 from Appendix A. At a first glance, we can notice notable dissimilarities between the 2 groups of EU countries, old and new MSs. Thus, the old EU-15 MSs registered the highest average limits scale (minimum and maximum) of almost all variables, namely, earnings, life expectancy, birth rate, public health allocations, healthy life for women and men, and perceived good and very good health—except for education components, and the lowest for poverty of elderly people (being almost at a half in the EU-15 group of countries—28.3%, compared to the EU-13 group—52%) (Appendix A, Table A1).

For an adequate comparison between the EU member states, we subjected the data, first, to the logarithmic procedure. Afterwards, we clustered the data in 2 separate panels, the old EU-15 countries, and the new ones, EU-13 (Appendix A, Table A1).

### 3.2. Methodology

The empirical research configured in this paper relies on 2 approaches to modelling panel data, namely, structural equation modelling (SEM) and network analysis through Gaussian graphical models (GGMs) for each group of countries (old and new EU countries). GGMs entail an undirected network of partial correlation coefficients (both positive and negative), graphically reflected through the absolute strengths (width and saturation of the edges between nodes), thus being a network model of conditional associations and avoiding spurious correlation. At the same time, SEM brings forward our research since it re-joins path analysis, factor analysis, and regression, thus allowing for specifying multiple causal associations between variables. In our research, SEM was used to develop causal understanding from observational data to entail not just the patterns and correlations between variables but also to identify and evaluate causal relationships [38]. Both SEM and GGM imply a variance–covariance matrix, aiming to identify how variables are related to each other, namely, the direct and indirect effects of one variable on another, having their origin in path analysis.

**Figure 4:**
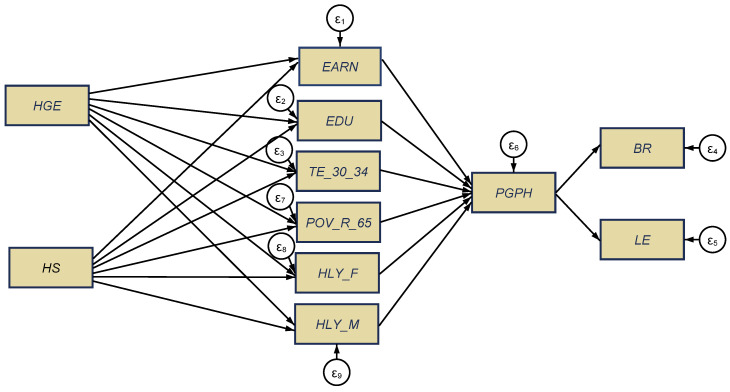
General configuration of health-aging interlinkages, structural equation modelling (SEM) model. Source: authors’ process in Stata 16 (StataCorp LLC, Texas, TX, USA).

By acknowledging that correlation does not necessarily imply causality [39,40], we configured an advanced *structural equation model (SEM) from the latent class analysis (Gaussian family*), since the SEM method “offers the potential for tentative causal inferences to be drawn when used with carefully specified and controlled designs” [41] (p. 253). SEM has allowed us to determine how each variable depends upon its immediate causal predecessors [42] and hence to perform an integrated assessment (direct, indirect, and total) of the health–aging conjunction, under the cumulative causation of selected social determinants. Given the implications of public health expenditures on people’s health condition, life expectancy, education [43,44], and fertility rate [45], with controversial results extracted from the literature, we appraised the general configuration of the cause and effect hypothesis SEM model as it is presented in Figure 4.

Subsequently, we investigated (i) the direct implications of public health support (reflected by *HGE* and *HS* variables) on selected economic and social variables (namely, as a cause of health conditions of older women and men, 65+ years aged, poverty rate of older people, education components, and income/earnings), (ii) the jointly cumulative effects of the above influences on the perceived health of people (as one of the long-term SDG indicators, Goal 3, the most affected by the COVID-19 pandemic crisis), (iii) and the overall impacts of all these cumulated factors on the aging dimensions, namely, birth rate and life expectancy (the last one being also a SDG indicator, included in Goal 3).

In order to reinforce overall interlinkages among variables through the use of models in the Gaussian family, we further applied the network analysis through Gaussian graphical models (GGMs).

*A Gaussian graphical model* for a random vector X = (X_1_, …, X_p_) is determined by a graph *G* on *p* nodes. The model comprises all multivariate normal distributions, $N(\mu ,\theta^{-1})$, whose inverse correlation matrix satisfies that $\theta_{jk}=0$, when {j,k} is not an edge in *G* [46]. The undirected graph G=(V,E) includes a vertex set V={1,…,p} as well as an edge set E⊂V×V [47] (p.3). Let $\Omega_{d}=(\omega_{ij,d})=\Sigma_{d}^{-1}$ for *d* = 1,2 be the precision matrix for $X={\left[x^{1},\ldots ,x^{n1}\right]}^{T}\in R^{n1xp}$ and $Y={\left[y^{1},\ldots ,y^{n2}\right]}^{T}\in R^{n2xp}$. *X* and *Y* denote the data matrices. The precision matrix (inverse covariance matrix) $\Omega =\Sigma^{-1}$ represents a GGM. A GGM associated with *X* is a graph, where the node set $V=\{x_{1},x_{2},\ldots \ldots ,x_{p}\}$ has p components and the edge set *E* such that any edge between *x_k_* and *x_j_* if and only if *x_k_* and *x_j_* are conditional, depending on all other variables. Similarly, a GGM associated with *Y* is also a graph [48] (p. 1).

Through the visual rendering, *GGMs* afford overall insights of the casual dependency and intensity of interaction among our items/variables [46,49]. The GGMs are designed for each considered panel, EU-13 and EU-15, through extended Bayesian information criterion (EBIC) with graphical lasso (EBICglasso).

In this framework, in concordance with our general objective and applied methodology, by reviewing the current state of the literature on the interplay between health and aging, we set the following research hypotheses (H):

**H1.**  There are strong direct implications of public health support on the health conditions of older people and other social indicators;

**H2.**  People’s good and very good perceived health is strongly influenced/shaped by the overall implications of public health support and other social dimensions;

**H3.**  The aging dimensions are significantly shaped by the compelling effects (total, direct, indirect) of health and other social dimensions.

## 4. Results and Discussions

### 4.1. The Results of Structural Equation Modelling (SEM)

**Figure 5:**
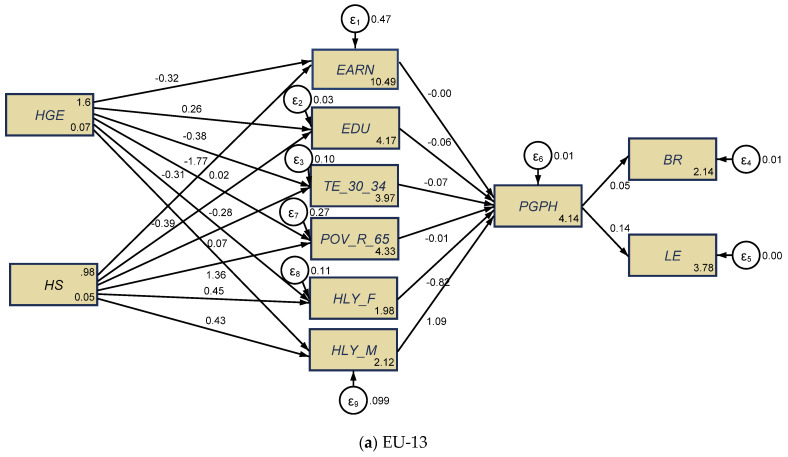

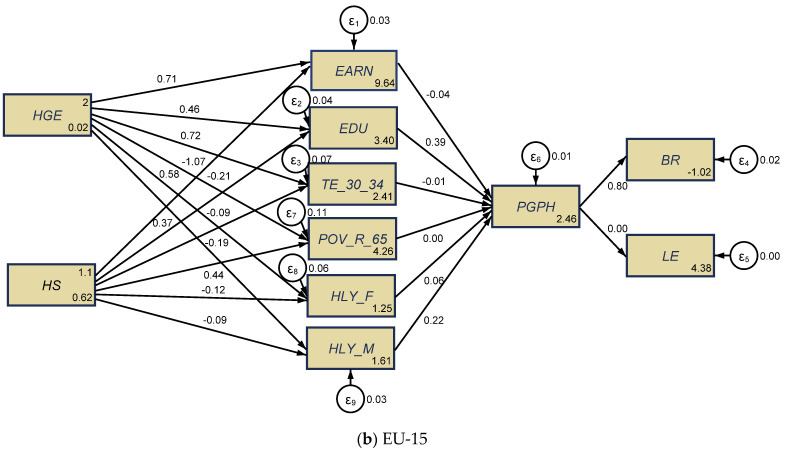
SEM results for the health–aging conjunction, the EU-13 (**a**), and the EU-15 (**b**), for the 1995–2017 lapse of time. Source: authors’ process in Stata 16 (StataCorp LLC, Texas, TX, USA).

In order to test our three research hypotheses, we configured two structural equation models, processed through the maximum likelihood estimator (MLE) for each of the two panels, namely, the new EU-13 countries (Figure 5a) and the old EU-15 MSs (Figure 5b). Detailed results of the SEM models for each group of EU countries are shown in Appendix A, Table A2.

The validity and robustness of SEM results was firstly checked by distinctive tests, namely, Cronbach’s alpha test for each panel, EU-13 and EU-15 (Appendix A, Table A3) (the alpha coefficient was 0.651, respectively 0.7618, for each considered panel, which points out a good reliability of the scale); the Wald test (Appendix A, Table A4); and the goodness-of-fit tests (Appendix A, Table A5), which enclose the coefficient of determination (CD) highlighting that over 50% (in the case of EU-13) and 57.3% (in the case of EU-15) of the variables have been shaped by the aging dimensions.

As regards direct implications of public health spending on the health conditions of older people and other social indicators (H1), we noticed that there was a causal relationship between government health expenditures and services and the health conditions of elderly people, since at the greatest extent, HGE induced positive influences on the variables considered for the EU-13 group (Figure 5a), while HS had positive direct influences; on the other hand, in the EU-15 countries, these influences were reversed (Figure 5b).

More specifically, for the EU-13 countries (Figure 5a, Table A5), healthy life of older people (65+) was favorably influenced/caused (to a statistically significant extent, *p* ≤ 0.001) by hospital services (*HS*), both for women (*HLY_F*) (the estimated coefficient was 0.447) and men (*HLY_M*) (the estimated coefficient was 0.428). By contrast, the health of older people was unfavorably affected/caused by public health expenditures (*HGE*) (to a statistically significant extent), with a far greater impact on older men (*HLY_M*) (the estimated coefficient was −0.391, *p* ≤ 0.001) than on (*HLY_F*) (the estimated coefficient was −0.307, *p* < 0.5). These results are similar to those obtained by Cristea et al. [8] (p. 11), meaning that, in the developing countries, “government health expenditure is not enough to ensure health promoting conditions for people of both genders aged 65”. However, in contrast with the findings of Cristea et al. [8], which were centered on the relationship between aging, public health support, and labor productivity, in the framework of an increased integration of the elderly group of working people (aged 55–64 years) on the labor market, our results were centered on the perceived health of people as good and very good (one of the 17th SDGs that target better health and well-being, namely, Goal 3 “Good health and well-being”), under the implications/causation of public health allocation and other socio-economic dimensions, with an overall impact on the aging credentials (birth rate and life expectancy). Conversely, poverty rate of older people (*POV_R_65*) was found to be lessened by government health allocation (*HGE*) (the estimated coefficient was −1.765, *p* ≤ 0.001) and augmented by public hospital services (*HS*) (the estimated coefficient was 1.357, *p* ≤ 0.001). Educational attainment plays a huge positive role in public health support (for *HGE* component) (levels 3–8) (*EDU*) (the estimated coefficient was 0.261, *p* ≤ 0.001), yet negative for tertiary education (*TE_30_34*) (the estimated coefficient was −0.383, *p* ≤ 0.001). As regards the HS component, the impact on education (levels 3–8) (*EDU*) was found to be negative (the estimated coefficient was −0.281, *p* ≤ 0.001). The influence of public health support on welfare (measured by *EARN*) was not relevant from a statistical point of view.

Regarding the old EU-15 countries (Figure 5b), the influence in terms of healthy life of older people (65+) was opposite to the results obtained for the new EU-13 group, being favorably influenced/caused by increased public health expenditures (*HGE*), both for women (*HLY_F*) (the estimated coefficient was 0.584, *p* ≤ 0.001) and men (*HLY_M*) (the estimated coefficient was 0.374, *p* < 0.05). Poor public hospital services (*HS*) (to a statistically significant extent, *p* ≤ 0.001) had a negative impact both on older men’s (*HLY_M*) (the estimated coefficient was −0.0896) and older women’s health (*HLY_F*) (the estimated coefficient was −0.124). These results are also similar to those obtained by Cristea et al. [8], as regards the direct implications of health allocation on healthy life of older women and men (over 65 years old) but focusing on labor productivity effects. Similar to the EU-13 group, the poverty rate of older people (*POV_R_65*) was found to be reduced by the government allocation to healthcare (*HGE*) (the estimated coefficient was −1.070, *p* ≤ 0.001), and increased by public hospital services (*HS*), but lower than in the EU-13 panel (the estimated coefficient was 0.441, *p* ≤ 0.001). These findings are supported by those obtained by Randel et al. [23], Von dem Knesebeck et al. [26], and Wicks-Lim and Arno [25], which have shown strong favorable connections between health and poverty. The education components were statistically and positively influenced also by the *HGE* component, both for level 3–8 (*EDU*) and for the tertiary education, as a share to the 30–34-year-old aged group (*TE_30_34*), and were negatively impacted by public *HS* for both variables of education (*EDU* and *TE_30_34*). Welfare (measured by *EARN*) was favorably influenced by public *HS* (the estimated coefficient was 0.713, *p* ≤ 0.001) and negatively affected by the health government spending (*HGE*) (the estimated coefficient was −0.209, *p* ≤ 0.001).

Thus, our first hypothesis, H1: “*There are strong direct implications of the public health financing on the health conditions of older people and other social indicators*”, was found to be partially fulfilled, with differentiation between new and old EU countries. Therefore, a causal relationship is attested between government health expenditure, hospital services, and the health conditions and well-being of elderly people (aged 65+).

Only education has a statistically significant and positive influence on the perceived health of people aged 16+ (*PGPH*, as long-term *SDG* indicator, regarding “good health and well-being”) (levels 3–8) (*EDU*) for the EU-15 group of countries (Figure 5b), under indirect influences of public healthcare financing (government spending and hospital services). This impact is similar to those obtained by Brunello et al. [32], Shields and Shooshtari [36], and Strulik [34]. For the EU-13 group (Figure 5a), *PGPH* was gender-differentiated, being under the positive impact of healthy life years of older men (*HLY_M*) (the estimated coefficient was 1.092, *p* ≤ 0.001), and under the direct negative impact of healthy life years of older women (*HLY_F*) (the estimated coefficient was −0.816, *p* ≤ 0.001) and tertiary education (*TE_30_34*) (the estimated coefficient was −0.0732, *p* < 0.05), jointly with the indirect implications of public healthcare support.

Thus, the second hypothesis, H2: “*People’s good and very good perceived health is strongly influenced/shaped by the overall implications of public health infrastructure and other social dimensions*”, was found to be partially fulfilled, with visible differences between the panels of the new and the old EU countries.

Finally, overall implications (total, direct, indirect) upon the aging dimensions (birth rate and life expectancy) under the synergies of health and other social dimensions were positive for both EU groups of countries. These impacts were, however, more visible for the old EU-15 countries (most of them being developed countries) as regards the influence on birth rate (the estimated coefficient was 0.799, *p* ≤ 0.001) than for the new EU-13 countries (mostly developing countries), which encountered a positive impact in terms of life expectancy (the estimated coefficient was 0.136, *p* ≤ 0.001), as Brunello et al. [32] and Strulik [34] have also proven.

Thus, the third hypothesis, H3: “*The aging dimensions are significantly shaped by the compelling effects (total, direct, indirect) of health and other social dimensions*”, was found to be fulfilled, being more pronounced for the old EU-15 countries. Therefore, a causal relationship was attested between government health expenditures, hospital services, people’s good and very good health conditions (including perceived ones) and their well-being, and the aging coordinates.

### 4.2. The Results of Gaussian Graphical Models (GGMs)

**Figure 6:**
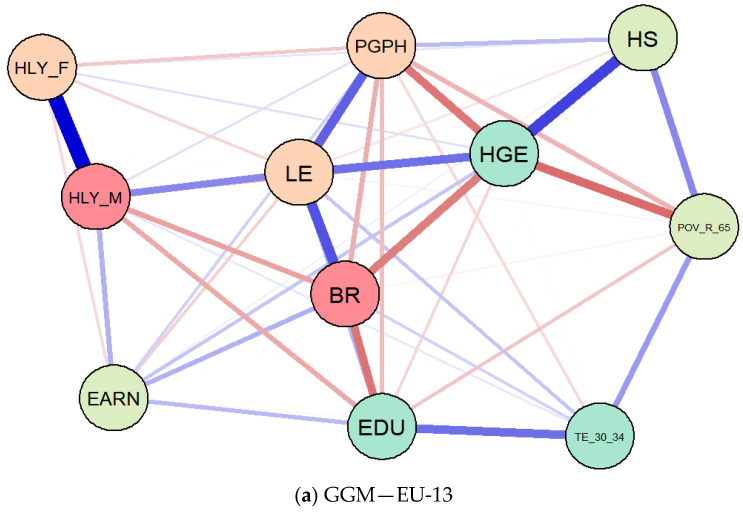

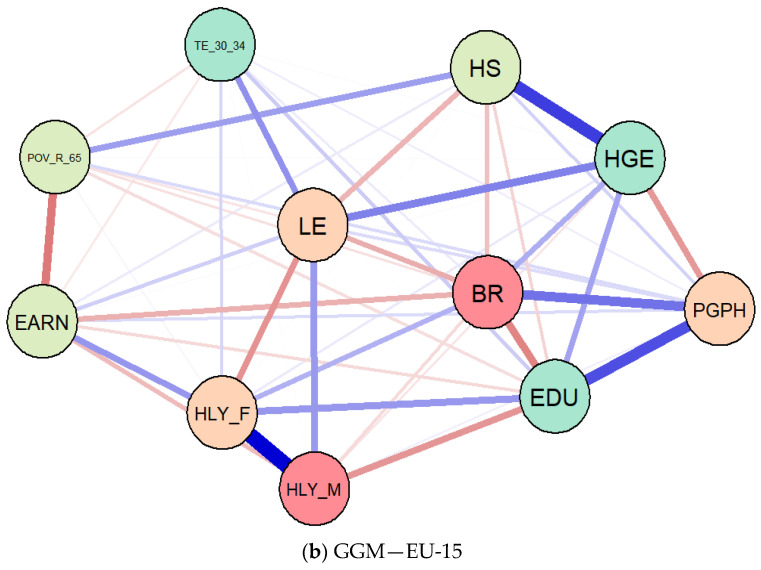
Results of Gaussian graphical models (GGMs), Extended Bayesian Information Criterion with graphical least absolute shrinkage and selection operator (EBICglasso) method, EU-13 (**a**) and EU-15 (**b**). Source: authors’ contribution in R programming version 3.6.3. (R Foundation, Free Software Foundation’s GNU General Public License, Boston, MA, USA).

The results of GGMs are entailed in Figure 6, being configured through the Extended Bayesian Information Criterion with graphical least absolute shrinkage and selection operator (EBICglasso) method. The main findings reinforce the results obtained through SEM—latent class analysis and our three hypotheses tested, and evidence significant interlinkages between the considered items.

Thereby, life expectancy (*LE*) was found to be positively connected with the perceived health of people aged 16+ (*PGPH*), both for EU-13 (Figure 6a) and EU-15 (Figure 6b) panels, but also with healthy life of older men (*HLY_M*), health government spending (*HGE*), and educational dimensions. As regards the healthy life years of older women (*HLY_F*), the association with life expectancy (*LE*) was unfavorable, both for the EU-15 group and EU-13. Birth rate (*BR*) was also positively connected with *PGPH* in terms of the EU-15 group, and negatively for the EU-13 countries. At the same time, there were also favorable linkages between poverty rate of older people (*POV_R_65*) and government health allocation (*HGE*) (in the case of EU-13), on the one hand, and they were found to be amplified by public hospital services (*HS*).

These findings are similar to previous results obtained after processing the SEM models and entail significant interlinkages and complex causal relationships between public health, well-being, and population aging across the EU MSs.

## 5. Conclusions

Against the background of the worldwide increasing significance of the aging phenomenon, with notable differences among countries/regions, and under consideration of its interlinkages with the economic and social life, we investigated in this paper the relationship between health, welfare, and aging in two distinctive groups of countries, the old (most of them developed countries) and the new EU member states (mostly developing countries) on the basis of historical data for the period 1995–2017. In order to draw out the overall impacts (direct, indirect, and total) among considered variables, the research methodology relied on the structural equation modelling (SEM) and Gaussian graphical model (GGM) techniques, which were applied for each distinctive group of EU countries.

On the basis of the literature findings and identified gaps, our results, assessed on three research directions/hypotheses, for each considered group, the EU-13 and EU-15, revealed that (i) there are direct implications of public health support (reflected through the health government expenditures and hospital services) in terms of the health of elderly people (women and men) and other social indicators (income, poverty rate of older people, education components, levels 3–8 and tertiary), with differences between the new and old EU countries, and with government expenses and education having a similar positive impact on lowering the poverty rate of elderly people (levels 3–8), and a negative impact considering public hospital services, more pronounced for the EU-13 MS than for the EU-15; (ii) there are joint cumulative effects of public health support and other social dimensions on the perceived health, good and very good, with notable differences between the new and the old EU countries; (iii) there are overall influences (total, direct, indirect) on the aging dimensions (namely, on the birth rate and life expectancy) under the compelling effects of health and other social dimensions, with a special focus on the increased birth rate within the EU-15 group of countries, and respective of life expectancy for the EU-13 panel.

Consequently, the main contributions of our results relate to the extent in which people’s health perception (as good and very good) is influenced by the public health allocations and other socio-economic dimensions (education, welfare, and healthy life), with overall impact on aging credentials (birth rate and life expectancy), distinctively in the case of old EU countries and new EU member states.

Thereby, the main implications of our findings relate to continuously updated specific/targeted policies and strategies developed by policymakers for each considered group of EU countries, as regards the interplay between public health support and aging, thus ensuring the prevention of a large part of the burden of disease, disability, and loss of well-being for the older population. These tailored policies and accurate measures can decisively increase older people’s health and well-being, and therefore should include guaranteed healthcare access at nearby locations for elderly people and additional public support for informal care-giving, centered on home care, self-care, and long-term palliative care; development of multifunctional systems in order to collect individuals’ health information and to rapidly assess their health conditions [7]; tackling ageist attitudes of communities, which have been more pronounced during the COVID-19 pandemic crisis [10,50]; sustaining and rethinking education for health customs and attitudes, especially for the developing EU-13 countries; well-oriented public government support for the improvement of health conditions of both women and men, especially for the developing EU-13 countries; and public hospital services for the EU-15 countries in order to create improved health-promoting conditions for the elderly people. Given the new challenges brought by the COVID-19 pandemic crisis, our findings may help other researchers on this topic, offering a comparative perspective of the existing situation before the coronavirus pandemic burst.

The main limitations of our research consist of a reduced availability of accurate data associated with some variables for the entire period of analysis (missing data), as well as in the unforeseen impact of the COVID-19 pandemic crisis, with manifold consequences, primarily on health and demographic indicators. Thus, in certain situations, the limitations relate to the results obtained that were inconclusive, with a lower degree of statistical significance of the estimated coefficients.

Further research should aim to search into each country of the considered EU groups, old and new, and to assess the inherent implications of the COVID-19 pandemic on public health support and company financing [51], healthy aging and well-being of older people, other associated social determinants of older employees (55–64-year-old group), such as work and job security, and urban developments.

## Data Availability

Data supporting reported results are compiled from the Eurostat database [18] and the dataset is available upon request.

## Appendix A

**Table A1:** Summary statistics for the EU-13 and EU-15 datasets, 1995–2017.

Variables	*n*	Mean	Standard Deviation	Minimum	Maximum
EU-13					
EDU	299	71.79532	14.59404	17.1	88
EARN	273	35414.8	43926.32	666.42	265212
LE	273	75.16593	3.220439	67.7	82.7
BR	299	10.07358	1.053504	7.6	15.2
TE_30_34	299	26.13512	12.49351	1	58.7
POV_R_65	140	19.98786	11.30065	4.1	52
HLY_F	161	7.165217	2.504128	2.7	14.2
HLY_M	161	7.017391	2.335047	3	13.5
HGE	289	4.975779	1.415306	1.8	7.9
HS	251	2.669323	0.6839117	0.8	5.1
PGPH	164	59.72256	9.925003	35	80.3
log_EDU	299	4.24058	0.29538	2.839078	4.477337
log_EARN	273	10.0157	0.9378622	6.50192	12.48829
log_LE	273	4.318788	0.0426822	4.215086	4.41522
log_BR	299	2.304702	0.1013955	2.028148	2.721295
log_TE_30_34	299	3.129542	0.5635107	0	4.07244
log_POV_R_65	140	2.811008	0.6461677	1.410987	3.951244
log_HLY_F	161	1.908461	0.3540796	0.9932518	2.653242
log_HLY_M	161	1.894291	0.3314473	1.098612	2.60269
log_HGE	289	1.558653	0.3164352	0.5877866	2.066863
log_HS	251	0.9468871	0.272641	-0.2231435	1.629241
log_PGPH	164	4.075466	0.1712212	3.555348	4.38577
N total	114				
EU-15					
EDU	341	63.13856	13.93617	19.3	82.3
EARN	317	46101.44	27095.49	1648.52	311052
LE	330	79.63242	1.790216	75.3	83.5
BR	345	11.01391	1.720831	7.6	16.7
TE_30_34	345	33.01696	11.05093	8.6	54.6
POV_R_65	165	15.07818	5.150729	4.7	28.3
HLY_F	192	9.878646	2.337115	5.2	16.8
HLY_M	192	9.684375	1.839294	6.2	15.7
HGE	345	6.488696	1.085938	3.7	8.9
HS	304	3.065461	1.436566	0	6.3
PGPH	196	70.79031	8.242352	45.9	84.5
log_EDU	341	4.113025	0.2762023	2.960105	4.410371
log_EARN	317	10.63519	0.4655938	7.407633	12.64772
log_LE	330	4.377168	0.0225438	4.32148	4.424847
log_BR	345	2.387325	0.1534143	2.028148	2.815409
log_TE_30_34	345	3.428878	0.3941481	2.151762	4.000034
log_POV_R_65	165	2.645185	0.3902959	1.547562	3.342862
log_HLY_F	192	2.26187	0.2422442	1.648659	2.821379
log_HLY_M	192	2.252649	0.1901278	1.824549	2.753661
log_HGE	345	1.855194	0.1758141	1.308333	2.186051
log_HS	287	1.015284	0.7525125	-2.302585	1.84055
log_PGPH	196	4.251958	0.1297185	3.826465	4.436751
*n* total	124				

**Table A2:** Detailed results of the SEM models, EU-13 and EU-15, 1995–2017.

Variables	(1)	(2)	Variables	(1)	(2)
	EU-13	EU-15		EU-13	EU-15
log_EARN			log_EDU_ATT		
log_HS	0.0192 (0.334)	−0.209 *** (0.0313)	log_HS	−0.281 ** (0.0891)	−0.0871 * (0.0349)
log_HGE	−0.324 (0.290)	0.713 *** (0.160)	log_HGE	0.261 *** (0.0773)	0.463 ** (0.178)
_cons	10.49 *** (0.404)	9.635 *** (0.288)	_cons	4.169 *** (0.108)	3.398 *** (0.321)
log_PGPH			log_TE_30_34		
log_EARN	−0.00199 (0.0165)	−0.0410 (0.0618)	log_HS	0.0663 (0.152)	−0.194 *** (0.0458)
log_EDU	−0.0612 (0.0642)	0.390 *** (0.0607)	log_HGE	−0.383 ** (0.132)	0.723 ** (0.233)
log_TE_30_34	−0.0732 * (0.0346)	−0.00606 (0.0375)	_cons	3.973 *** (0.183)	2.412 *** (0.421)
log_POV_R_65	−0.0131 (0.0164)	0.00262 (0.0217)	log_POV_R_65		
log_HLY_F	−0.816 *** (0.0971)	0.0559 (0.102)	log_HS	1.357 *** (0.253)	0.441 *** (0.0579)
log_HLY_M	1.092 *** (0.113)	0.217 (0.123)	log_HGE	−1.765 *** (0.220)	−1.070 *** (0.295)
_cons	4.139 *** (0.331)	2.458 *** (0.564)	_cons	4.331 *** (0.305)	4.260 *** (0.532)
log_HLY_F			log_HLY_M		
log_HS	0.447 ** (0.164)	−0.124 ** (0.0415)	log_HS	0.428 ** (0.153)	−0.0896 ** (0.0320)
log_HGE	−0.307 * (0.143)	0.584 ** (0.211)	log_HGE	−0.391 ** (0.132)	0.374 * (0.163)
_cons	1.976 *** (0.199)	1.248 ** (0.381)	_cons	2.115 *** (0.184)	1.613 *** (0.294)
log_BR			log_LE		
log_PGPH	0.0472 (0.0420)	0.799 *** (0.0920)	log_PGPH	0.136 *** (0.0173)	0.00374 (0.00748)
_cons	2.136 *** (0.171)	−1.020 ** (0.392)	_cons	3.784 *** (0.0706)	4.378 *** (0.0318)
var(e.log_EARN)			var(e.log_PGPH)		
_cons	0.473 *** (0.0626)	0.0326 *** (0.00414)	_cons	0.00996 *** (0.00132)	0.00582 *** (0.000739)
var(e.log_EDU_ATT)			var(e.log_TE_30_34)		
_cons	0.0336 *** (0.00444)	0.0404 *** (0.00513)	_cons	0.0973 *** (0.0129)	0.0696 *** (0.00884)
var(e.log_POV_R_65)			var(e.log_HLY_F)		
_cons	0.271 *** (0.0359)	0.111 *** (0.0141)	_cons	0.114 *** (0.0152)	0.0570 *** (0.00725)
var(e.log_HLY_M)			var(e.log_BR)		
_cons	0.0986 *** (0.0131)	0.0340 *** (0.00432)	_cons	0.00523 *** (0.000693)	0.0179 *** (0.00227)
var(e.log_LE)					
_cons	0.000887 *** (0.000117)	0.000118 *** (0.0000150)			
*n*	114	124			

Note: Standard errors in parentheses, * *p* < 0.05, ** *p* < 0.01, *** *p* < 0.001. Source: authors’ contribution in Stata 16 (StataCorp LLC, Texas, TX, USA).

**Table A3:** Cronbach’s alpha for SEM, EU-13 and EU-15 models, 1995–2017.

Variables	SEM 1—EU-13			SEM 2—EU-15		
	Observation	Sign	Alpha	Observation	Sign	Alpha
Log_EARN	273	+	0.5205	317	+	0.7413
Log_PGPH	164	+	0.4524	196	+	0.7120
Log_EDU	299	−	0.5272	341	+	0.7355
Log_TE_30_34	299	+	0.5928	345	+	0.7069
Log_POV_R_65	140	+	0.5217	165	−	0.7360
Log_HLY_F	161	+	0.4009	192	+	0.7079
Log_HLY_M	161	+	0.3722	192	+	0.7146
Log_BR	299	−	0.6601	345	+	0.7628
Log_LE	273	+	0.4554	330	+	0.7763
Log_HS	251	+	0.5603	287	−	0.7586
Log_HGE	289	−	0.6240	345	−	0.8098
Total scale			0.6510			0.7618

Source: authors’ contribution in Stata 16 (StataCorp LLC, Texas, USA).

**Table A4:** Wald tests for equations associated with the SEM models, 1995–2017.

Variables	SEM 1—EU-13			SEM 2—EU-15		
	Chi^2^	*df*	*p*-Value	Chi^2^	*df*	*p*-Value
Log_EARN	1.71	2	0.4257	45.00	2	≤0.001
Log_PGPH	183.94	6	≤0.001	239.38	6	≤0.001
Log_EDU	13.75	2	≤0.001	7.43	2	0.0244
Log_TE_30_34	10.53	2	0.0052	18.04	2	≤0.001
Log_POV_R_65	65.79	2	≤0.001	68.43	2	≤0.001
Log_HLY_F	7.97	2	0.0186	9.50	2	0.0086
Log_HLY_M	10.68	2	0.0048	7.90	2	0.0192
Log_BR	1.27	1	0.2606	75.34	1	≤0.001
Log_LE	61.61	1	≤0.001	0.25	1	0.6170
H0: all coefficients excluding the intercepts are 0. We can thus reject the null hypothesis for each equation, with limitations on EARN and BR for EU-13 and LE for EU-15.						

Source: authors’ contribution in Stata 16 (StataCorp LLC, Texas, USA).

**Table A5:** Goodness-of-fit tests for the SEM models, 1995–2017.

Explanations	SEM 1—EU-13	SEM 2—EU-15
Likelihood ratio		
Model vs. saturated chi^2^_ms (15)	573.975	754.533
*p* > chi^2^	≤0.001	≤0.001
Baseline vs. saturated chi^2^_bs (24)	828.606	1080.730
*p* > chi^2^	≤0.001	≤0.001
Information criteria		
AIC (Akaike’s information criterion)	−365.186	−1137.624
BIC (Bayesian information criterion)	−261.210	−1030.453
Baseline comparison		
CFI (comparative fit index)	0.803	0.898
TLI (Tucker–Lewis index)	0.407	0.515
Size of residuals		
CD (coefficient of determination)	0.508	0.573

Source: authors’ contribution in Stata 16 (StataCorp LLC, Texas, USA).
